# Occurrence of Liver Damage and Obstetric Outcomes in Pregnant Women Diagnosed with Pruritus during Pregnancy: A Retrospective Study

**DOI:** 10.1155/2022/5913712

**Published:** 2022-09-14

**Authors:** Ena Pavelic, Vladimir Blagaic, Paulo Zekan, Petra Glad Stritof, Mara Bebek, Josko Bilandzic, Milan Pavlovic, Mihovil Herceg

**Affiliations:** ^1^Health Center Zagreb East, Svarcova 20, Zagreb 10000, Croatia; ^2^Department of Obstetrics and Gynecology, Clinical Hospital Sveti Duh, Sveti Duh 64, Zagreb 10000, Croatia; ^3^Department of Physics, Faculty of Natural Sciences and Mathematics, University of Zagreb, Bijenicka Cesta 32, Zagreb 10000, Croatia

## Abstract

**Aim:**

A retrospective study of the occurrence of liver damage and obstetric outcomes in pregnant women diagnosed with pruritus.

**Methods:**

The following parameters were monitored in patients: aspartate aminotransferase (AST), alanine aminotransferase, gamma-glutamyl transferase, bilirubin (direct and total), hemoglobin, platelets, serum bile acid level, age of pregnant women, parity, pregnancy weight gain, birth weight, and gestational age at delivery. A total of 107 patients were included during a five-year period (2016–2020) and classified into three groups. Group A included 17 pregnant women with pruritus without elevated liver enzymes and bilirubin. Group B included 50 pregnant women with pruritus, elevated liver enzymes, and bilirubin. Group C included 40 pregnant women with pruritus and elevated bile acids (regardless of liver enzyme levels).

**Results:**

The groups did not significantly differ in patients' age and parity, but there was a statistically significant between-group difference in weight gain during pregnancy. The values of AST, ALT, GGT, LDH, and direct bilirubin were the highest in group B, and serum bile acids were expectedly the highest in group C. There was no statistically significant variation in the onset of labor and mode of delivery between groups. However, groups significantly differed in gestational age at delivery, newborn birthweight, and pregnancy prolongation from the onset of pruritus to delivery.

**Conclusion:**

Further study is needed to assess the pathophysiologic mechanisms underlying intrahepatic cholestasis of pregnancy as well as any significant liver damage associated with pregnancy.

## 1. Introduction

Pruritus is an itching of the skin that is associated with allergic reactions and also liver disease with elevated bile acid levels. The exact mechanism of the pruritus onset is still unknown, but the occurrence of the pruritus is thought to be based on the action of histamine. However, the review published in 2018 indicates the connection between the occurrence of pruritus and the action of some other mediators, such as 5-hydroxytryptamine, opioid peptides, and proteases [1]. Furthermore, pruritus may be an alarming symptom in pregnancy since it may be the sole manifestation of intrahepatic cholestasis during pregnancy–condition that is harmless for pregnant women but is associated with an increased risk of stillbirth [2]. Interestingly, 23–38% of women report pruritus during pregnancy, and 2% report even severe pruritus [3].

In the most recent literature, liver disease associated with pregnancy is clearly divided into two main groups: liver disease associated with pregnancy and primary liver disease concurrent with pregnancy. Liver diseases associated with pregnancy can be classified into those occurring in early pregnancy (hyperemesis gravidarum) and those occurring in late pregnancy (acute fatty liver of pregnancy-AFLP, preeclampsia/eclampsia, hemolysis, elevated liver enzymes, low platelets (HELLP) syndrome, liver rupture/infarction, and intrahepatic cholestasis of pregnancy) [4]. Primary liver diseases concurrent with pregnancy are hepatitis B, hepatitis C, autoimmune hepatitis, nonalcoholic fatty liver (NAFLD), cirrhosis, and portal liver hypertension [5].

Numerous studies investigated different forms and degrees of liver disease in pregnancy, and some of them found a possible etiology leading to disease. Those studies also included reviews of preexisting liver disease and damage even before pregnancy, which resulted in the impaired liver function in pregnancy itself [6–9].

The aim of this paper is to present obstetric outcomes and the occurrence of liver damage, in pregnant women diagnosed with pruritus during pregnancy. The goal is to cover the full spectrum of liver damage from nonspecific liver lesions to severe forms of intrahepatic cholestasis.

## 2. Methods

The data were collected retrospectively from the hospital records. The patients who gave birth in the five-year period from 2016 to 2020 and had a history of pruritus in the current pregnancy were included in the study. The patients diagnosed with hyperemesis gravidarum, fatty liver of pregnancy, preeclampsia, eclampsia, or HELLP syndrome were excluded from the study. The patients were divided into the three groups. Group A included pregnant women with pruritus but without elevated liver enzymes or bile acids; group B included pregnant women with pruritus, elevated liver enzymes, and normal levels of bile acids; and group C included pregnant women with pruritus and elevated bile acids (regardless of liver enzyme levels). Elevated liver enzymes were defined as AST or ALT levels at least twice above the upper border of the reference range of a hospital laboratory (reference ranges are: AST 8–30 U/L and ALT 8–36 U/L). Elevated serum bile acids were defined as being above 10 *μ*mol/L.

The variables that were studied are as follows: biochemical and hematological parameters (AST, ALT, GGT, bilirubin (direct and total), serum bile acid levels, hemoglobin, platelets, LDH), epidemiological and obstetric characteristics (age of pregnant women, parity, weight gain of pregnant women during pregnancy), obstetric outcomes (week of gestation at birth–completed weeks, prolongation of pregnancy from the diagnosis of pruritus to delivery–in days, the onset of labor (spontaneous, induced, elective cesarean section), mode of delivery (vaginal, cesarean section), newborn birth weight and presence of serious complications during pregnancy or labor (stillbirth, postpartum hemorrhage or postpartum hysterectomy) and therapy given to treat pruritus.

Since patients in group C fulfilled the criteria for the diagnosis of intrahepatic cholestasis of pregnancy (ICP), we further separately analyzed biochemical and hematologic parameters and obstetric outcomes in high- and low-risk ICP. Patients with bile acids above 40 *μ*mol/L were included in the high-risk subgroup.

Microsoft Excel® was used for data collection. Numerical variables were statistically analyzed using an ANOVA and the post-hoc *t*-test. Categorical variables were analyzed with the chi-square test with a post-hoc analysis of adjusted residuals. (RStudio, Version 1.1.383-© 2009–2017 RStudio, Inc.). The data were given as a mean with a 95% confidence interval (CI). *p*-value less than 0,05 was considered statistically significant.

The study was approved by the Institutional Review Board (Ethics Committee) of the hospital.

## 3. Results

Out of 13 932 pregnant women who gave birth in the five-year period, 107 of them were included in the study. Groups A, B, and C consisted of 17, 50, and 40 pregnant women, respectively.

Epidemiological and obstetric characteristics of the studied sample are shown in Table 1.

Three groups did not significantly differ in patients' age and parity, but there was a statistically significant between-group difference in weight gain during pregnancy.

Biochemical and hematological parameters of the studied sample are shown in Table 2.

Values of AST, ALT, GGT, direct bilirubin, LDH, and serum bile acids varied significantly between the three groups. Values of AST, ALT and direct bilirubin were the highest in group B. Post-hoc analysis revealed that the values were significantly different between A vs. B and B vs. C. However, the former is biased due to the group's selection criteria. GGT and LDH were also the highest in group B. However, post-hoc multiple comparisons with Student's *t*-tests did not reveal any statistically significant variation between groups. The value of serum bile acids was expected to differ significantly between groups because of the initial group selection criteria. The values of all other hematologic and biochemical parameters did not significantly differ between groups.

Obstetric outcomes are shown in Table 3.

There was no statistically significant variation in the onset of labor and mode of delivery between groups. However, groups significantly differed in gestational age at delivery, newborn birthweight, and pregnancy prolongation from the onset of pruritus to delivery. The patients in group B gave birth at the earliest gestational age; they had the shortest prolongation period, and their newborns had the smallest birthweight. Additionally, there were no premature newborns in group A, but 46% and 40% of infants were premature in groups B and C, respectively (out of which 22% and 25% were born before the 34^th^ gestational week).

There was a significant difference in prescribing ursodeoxycholic acid within the three groups. The post-hoc analysis of adjusted residuals revealed that the patients in groups C were treated significantly more than the two other groups. 0%, 8%, and 30% of patients were treated in groups A, B, and C, respectively. All treated patients were given 1000–1500 mg of ursodeoxycholic acid, divided into two to three daily doses. In group C, one pregnant woman had BRIC (benign recurrent intrahepatic cholestasis), and therefore was taking ursodeoxycholic acid from the beginning of pregnancy, while all other patients started taking it after the onset of pruritus. For all three groups, pregnancy prolongation was not statistically significant in patients who were taking ursodeoxycholic acid therapy compared to those patients who were not taking that therapy. However, analyzing group C separately revealed that pregnancy prolongation was statistically significant in patients who were taking ursodeoxycholic acid vs. those who were not.

The separate analysis of group C (Table 4), i.e., the. Low-vs. high-risk group, revealed that high-risk patients had significantly higher values of LDH and platelets. Regarding obstetric outcomes, they gave birth at an earlier gestation and had newborns of lower birth weight. The treatment rate in a high-risk group was 39% compared to 29% in a low-risk group. All other parameters did not significantly differ between the two subgroups.

Serious complications occurred in two patients in group B. The first gave birth to a stillborn male child in the 36^th^ gestational week. The second had a massive placental abruption requiring urgent cesarean section, followed by postpartum hemorrhage requiring hysterectomy.

Additionally, three patients from the group C had a chronic liver disease that had been diagnosed before pregnancy, i. e., one had chronic hepatitis B, one had chronic hepatitis C, and one had BRIC. Two patients with hepatitis B and C were in the well-maintained chronic hepatitis phase with satisfactory liver function before pregnancy. The patient with BRIC has a congenital narrowing of the intrahepatic bile ducts since birth and has been using ursodeoxycholic acid therapy since she was 13 years old. However, the normal anatomy of intra and extrahepatic bile ducts is mandatory for the diagnosis of BRIC, so the patient may have been misdiagnosed with BRIC before pregnancy.

## 4. Discussion

The most striking result of the aforementioned data is that patients diagnosed with pruritus and elevated liver enzymes but normal values of bile acids had the most significant liver damage and the worst obstetric outcomes. This is surprising since the rise in bile acids is known to be associated with poor perinatal outcomes, but not rise in liver enzymes (other than acute fatty liver from pregnancy).

The groups did not significantly differ in the patients' parity. Still, the proportion of multiparous women was the highest in group C (62%). In addition, following the medical records of 51 nulliparous women from our sample, we found that almost 12% of them had a recurrent episode of pruritus or ICP in a subsequent pregnancy. This matches well with the results of one retrospective cohort study, which found that 16.6% of multiparas had intrahepatic cholestasis in previous pregnancies. The study included 3,710 pregnant women, 32 of whom were diagnosed with intrahepatic cholestasis in pregnancy [10].

Furthermore, the groups differed significantly in the values of AST and ALT. The A vs. B variation is due to the selection bias, as it was previously mentioned. However, the poorer liver function in group B patients compared to group C patients is interesting. We believe there may be a few explanations for that. Firstly, the onset of pruritus can precede the rise in serum bile acids for several weeks. Hence, some of the group B patients may have had undiagnosed ICP because they gave birth before the rise in serum bile acids. Thus, they may have been inappropriately been classified into group B rather than group C. Secondly, the rise in AST and ALT is not a diagnostic criterion for ICP, and some patients with ICP might have normal values of AST and ALT. Thirdly, group B patients might have been undiagnosed with the liver disease associated with pregnancy since they gave birth before the full onset of the disease [11].

Moreover, obstetric outcomes were significantly different between groups. However, shorter prolongation intervals and lower birth weight are associated with an earlier gestation at delivery, so this might be a cofounding factor. The post-hoc analysis revealed that the patients in groups B and C delivered earlier than the patients in group A. On the other hand, the patients in group A had the lowest rate of spontaneous onset of labor, although this was statistically insignificant. This might be in accordance with the previous studies showing that ICP increases the risk of spontaneous preterm labor. According to the retrospective cohort study published in the Lancet in 2019, spontaneous preterm birth occurred more frequently in ICP patients compared to the healthy pregnant population (13.4% vs. 4%; OR 3.47, 95% CI 3.06–3.95, iatrogenic preterm birth OR 3.65%, 95% CI 1.94–6.85) [12].

The separate analysis of high- and low-risk subgroups of group C revealed that the high-risk patients delivered earlier compared to the low-risk patients. Regarding the proper timing of delivery in patients with ICP, there is a retrospective cohort study conducted in Denmark that examined whether women with intrahepatic cholestasis in pregnancy (ICP) and total serum bile acids (TBA) ≥ 40 *μ*mol/L have a higher risk of feto-maternal complications when childbirth is induced in the 37^th^ week of pregnancy, compared with the term induction of labor in women with low-risk ICP. The study included 16,185 women who gave birth at Roskilde University Hospital between 2006 and 2011. In pregnant women with high-risk ICP (TBA ≥ 40 *μ*mol/l) labor was induced at 37 weeks of gestation, while in pregnant women with low-risk ICP (TBA < 40 *μ*mol/l) labor was induced at term. The results showed that the rate of feto-maternal complications was 1.2% (95% CI 1.05–1.39%) and for the high-risk ICP, 0.4% (95% CI 0.27–0, 46%). The *abovementioned* results indicate that the early induction of labor in the 37^th^ week of pregnancy seems justified in high-risk cases of ICP because, in addition to shortening the gestational age by 9 days and reducing the birth weight of newborns by 296 g, the induction of labor was not accompanied by noticeable poor outcomes for pregnant women and their fetuses. The earlier induction of labor was also favored in the group with severe ICP due to the expected large reduction in the risk of stillbirth [13].

With respect to treatment, group C was given UDCA significantly more often. Although we did not compare the intensity of pruritus between groups, it may be justified to believe that ICP patients had the most intense pruritus and consequently received treatment at the highest rate. This seems reasonable, since UDCA is known to relieve pruritus but does not improve perinatal outcomes. Furthermore, even the decline in bile acid levels after treatment does not decrease the risk of stillbirth. In a double-blind, multicenter, randomized placebo-controlled trial conducted by Chapell et al. and published in the Lancet in 2019, some maternal benefits of UCDA usage in ICP patients were reported. However, fetal/neonatal outcomes were not improved [14]. Furthermore, in the UDCA group, maternal itch score and alanine aminotransferase (ALT) improved, while the mean maternal bile acid concentration was slightly higher. However, the small improvement in itch score, though statistically significant, was unlikely to be clinically important. Fetal/neonatal outcomes were not improved with UDCA treatment compared with placebo: the composite primary outcome of perinatal death, preterm delivery, or neonatal intensive care unit (NICU) admission was 23 versus 27 percent (risk ratio (RR) 0.85, 95% CI 0.62–1.15) [11, 14].

The complications of pregnancy, which included stillbirths and postpartum hemorrhage leading to hysterectomy and were found only in group B. However, we believe our study was not powered enough to adequately assess differences in complication rates between groups. There is a high chance that the complications which occurred in group B were not a consequence of elevated liver enzymes. The already mentioned systematic review conducted by Ovadia et al., published in the Lancet in 2019, which included 5,557 cases of intrahepatic cholestasis in pregnancy and 165,136 controls (3.37% of intrahepatic cholestasis in the sample), confirmed the fact that the risk of stillbirth was increased in pregnant women with intrahepatic cholestasis, especially at serum bile acid values >/ = 100 *µ*mol/L (0.91% versus 0.32%; OR 1.46, 95% CI 0.73–2.89) [12]. Also, one prospective clinical study lasting 8 years was able to point out the fact that neonatal respiratory distress syndrome occurs more frequently in children of mothers who had intrahepatic cholestasis during pregnancy. In the same study, it was found that postpartum hemorrhage was twice as common in mothers with intrahepatic cholestasis [15].

## 5. Conclusion

This study presented obstetric outcomes and the occurrence of liver damage in pregnant women diagnosed with pruritus. Although the worst outcomes were expected in patients diagnosed with intrahepatic cholestasis of pregnancy, it seems that even worse outcomes occurred in patients with pruritus, elevated liver enzymes, and normal values of serum bile acids. Therefore, further study is needed to assess the pathophysiologic mechanisms underlying intrahepatic cholestasis of pregnancy as well as any significant liver damage associated with pregnancy.

## Figures and Tables

**Table 1:** Epidemiological and obstetric characteristics. Age and weight gain are given as a mean with 95%, CI while parity is given as an absolute number and ratio. ^*∗*^Statistically significant.

	Group A	Group B	Group C	*p*-value
Age	31.8 (28.9–34.6)	31.4 (29.6–33.2)	32.2 (21.2–33.2)	0.776
Parity				0.463
0	10 (59%)	26 (52%)	15 (38%)	
1	3 (18%)	15 (30%)	15 (38%)	
>1	4 (23%)	9 (18%)	10 (24%)	
Weight gain	13.8 (11.6–16.0)	9.8 (8.1–11.5)	10.2 (8.0–12.3)	0.046^*∗*^

**Table 2:** Values of biochemical and hematologic parameters. Data are given as a mean with 95% CI. ^*∗*^Statistically significant.

	Group A	Group B	Group C	*p*-value (anova)	*p*-value (A vs. B)	*p*-value (A vs. C)	*p*-value (B vs. C)
AST	22.53 (17.9–27.1)	168.4 (118.7–218.0)	88.1 (52.0–124.3)	0.0005^*∗*^	0.0007^*∗*^	0.31013	0.02428^*∗*^
ALT	18.6 (13.1–24.1)	256.1 (192.7–319.5)	142.0 (80.6–203.4)	<0.0001^*∗*^	<0.0001^*∗*^	0.083	0.020^*∗*^
GGT	16.5 (10.3–22.7)	37.3 (24.7–49.9)	23.1 (15.2–30.9)	0.0434^*∗*^	0.089	0.167	1
Total bilirubin	9.5 (6.9–12.0)	11.1 (7.3–14.9)	9.8 (8.0–11.6)	0.759	1	1	1
Direct bilirubin	2.3 (1.4–3.2)	6.9 (4.6–9.2)	2.6 (1.7–3.5)	0.00105^*∗*^	0.017^*∗*^	1	0.003^*∗*^
LDH	161.3 (126.9–195.9)	242.8 (205.4–280.2)	188.2 (147.0–229.5)	0.0246^*∗*^	0.053	1	0.117
Hemoglobin	105.3 (88.7–122.0)	102.7 (91.8–113.6)	93.9 (76.6–111.2)	0.548	1	1	1
Platelets	203.2 (150.1–256.2)	232.3 (198.9–265.8)	199.7 (152.3–247.0)	0.439	1	1	0.69
Serum bile acids	1.0 (0–2)	1.1 (0.3–1.9)	54.2 (27.6–80.9)	<0.0001^*∗*^	1	0.00078^*∗*^	<0.0001^*∗*^

**Table 3:** Obstetric outcomes. Data are given as a mean with 95% CI for all parameters except onset of labour, mode of delivery, serious complications, and treatment, which are given as absolute numbers with a ratio. Data given as a mean was analyzed using the ANOVA with post-hoc Student's *t*-tests + and others using the *X*2 test with post-hoc analysis of adjusted residuals $. Serious complications were not statistically analyzed. The *p*-value of *X*^2^ for the onset of labour and mode of delivery was insignificant, so post-hoc analysis was not performed. ^*∗*^Statistically significant.

	Group A	Group B	Group C	*p*-value (anova/*X*^2^)	*p*-value (A vs. B)	*p*-value (A vs. C)	*p*-value (B vs. C)
Gestation at delivery^+^	39.2 (38.7–39.7)	36.8 (36.1–37.5)	37.0 (36.5–37.5)	<0.0001^*∗*^	<0.0001^*∗*^	0.00051^*∗*^	1
Prolongation of pregnancy (in days)^+^	7.4 (4.0–10.8)	6.5 (4.5–8.5)	14.9 (9.9–20.0)	0.003^*∗*^	0.9	0.064	0.003^*∗*^
Onset of labour^$^				0.773			
Spontaneous	0 (0%)	4 (8%)	4 (10%)				
Induced	14 (82%)	37 (74%)	29(73%)				
Elective cesaren section	3 (18%)	9 (18%)	7 (17%)				
Mode od delivery^$^				0.463			
Vaginal	11 (65%)	30 (60%)	29 (73%)				
Cesaren section	6 (35%)	20 (40%)	11 (27%)				
Newborn birth weight^+^	3488 (3313–3663)	2966 (2756–3175)	3115 (2953–3277)	0.0101^*∗*^	0.0075^*∗*^	0.1079	0.7623
Serious complications							
Stillbirth	0	1	0				
Postpartal hemorrhage	0	1	0				
Peripartal hysterectomy	0	1	0				
Treatment^$^				0.003^*∗*^			
Urodeoxycholic acid	0 (0%)	4 (8%)	12 (30%)	Adjusted residuals *p*-value: A–0.36; B–0.35; C–0.001^*∗*^			
No treatement	17 (100%)	46 (92%)	28 (70%)				

**Table 4:** Subgroup analysis of low- vs. high-risk subgroups of group C. Data are given as a mean with 95% CI, except for treatment, which is given as a ratio. All variables except treatment were analyzed using a Student's *t*-test. Treatment was not statistically analyzed. ^*∗*^Statistically significant.

	Low-risk ICP (*n* = 24)	HIGH-RISK ICP (*n* = 13)	*p*-value
AST	74.9	112.7	0.333
ALT	108.8	203.5	0.203
GGT	22.6	23.8	0.864
Total bilirubin	9.1	11.1	0.340
Direct bilirubin	2.0	3.8	0.086
Ldh	155.6	248.4	0.048^*∗*^
Hemoglobin	84.0	112.3	0.081
Platelets	167.5	259.1	0.045^*∗*^
Gestation at delivery	37.6	36.0	0.001^*∗*^
Newborn birth weight	3322	2615	0.0002^*∗*^
Treatment with urodeoxycholic acid	29%	39%	

## Data Availability

The data are available upon request to the corresponding author.
